# Does financial literacy influence preventive health check-up behavior in Japan? a cross-sectional study

**DOI:** 10.1186/s12889-022-14079-8

**Published:** 2022-09-08

**Authors:** Sumeet Lal, Trinh Xuan Thi Nguyen, Abdul-Salam Sulemana, Mostafa Saidur Rahim Khan, Yoshihiko Kadoya

**Affiliations:** School of Economics, Hiroshima University, 1-2-1 Kagamiyama, Higashihiroshima, Hiroshima, 7398525 Japan

**Keywords:** Financial education, Financial literacy, Japan, Health behavior, Health investment, Preventive health check-up, Rationality explanation

## Abstract

**Background:**

General health check-ups are an important element of healthcare, as they are designed to detect diseases, thereby reducing morbidity and mortality. Recent studies have found that financial literacy promotes preventive healthcare usage and reduces risky health behaviors such as smoking, lack of exercise, and gambling. Based on this evidence, we hypothesize that financial literacy, as a rational decision-making tool, is positively associated with health check-up behavior in Japan.

**Methods:**

We extracted data on financial literacy, the main explanatory variable of this study, from the 2010 wave of the Preference Parameter Study (PPS) of Osaka University. Data on health check-up behavior as a dependent variable, along with control variables, were obtained from the 2011 PPS wave. Our sample focused on Japan’s middle-aged working population (40–64 years), and we applied probit regressions to test our hypothesis.

**Results:**

Our final sample size was 2,208 participants after merging the two datasets. Descriptive statistics show that respondents had moderate financial literacy (mean = 0.62, SD = 0.33), low financial education (mean = 0.17, SD = 0.38), and low participation (mean = 31.75%, SD = 46.56%) in the health check-up. The probit regression analysis showed that financial literacy is insignificantly associated with health check-up behavior in Japan (coefficient = -0.0229; 95% CI: -0.2011—0.1551; p-value = 0.801). However, demographic factors such as being male (coefficient = -0.2299; 95% CI: -0.3649—-0.0950; *p*-value = 0.001), older (coefficient = 0.0280; 95% CI: 0.0188 – 0.0371; *p*-value = 0.000), and married (coefficient = 0.3217; 95% CI: 0.0728 – 0.5705; p-value = 0.011), as well as risky health behavior such as smoking (coefficient = -0.2784; 95% CI: -0.4262—-0.1305; *p*-value = 0.000) are significantly related to health check-up behavior.

**Conclusions:**

Our results suggest that financial literacy insignificantly motivates people to behave rationally and understand the value of health check-ups as a tool for sustainable health.

## Introduction

Financial literacy is the ability to understand and effectively use financial skills to make sound financial decisions. Financial literacy helps people make better informed economic and financial decisions and commit to more rational health behaviors; financially literate people have a lower likelihood of smoking and addiction to gambling, and exercise more frequently [1–6]. Recently, Chan et al. [7] found a positive relationship between financial literacy and preventative healthcare in the US. These studies argue that financial literacy, being a tool for rational decision making, helps people think and behave in a rational way, thereby reducing engagement in activities harmful to health. These findings encourage us to explore health check-up, another potential health promoting behavior, from the perspective of rationality. There is much evidence substantiating health checks as a way to prevent illness, maintain good health, and alleviate many of the problems associated with aging at a lower cost [8, 9]. As a result of the derived health and economic benefits of health check-up, the rationality explanation of previous studies may be plausible for health check-up as well. However, unlike other health promoting behaviors that have clear outlined benefits, several other studies have indicated that health checks are a complex and controversial issue [10–12].

General health checks are a common element of healthcare in Japan, as they aim to detect early disease risk factors to reduce morbidity and mortality [13]. In 2008, the Ministry of Health, Labor, and Welfare (MHLW) developed Specific Health Check-ups (SHC) and Specific Health Guidance programs that mandated all Japanese health insurers to provide health check-up programs to enrollees aged 40‒74 years and their dependents [14]. However, a gap exists between the government’s goals and actual participation [15]. For example, under the SHC program, only 46.2% of the target individuals received check-ups in 2014, which was far from the goal of 70%, set by the MHLW [16]. Consequently, the act of determining whether or not to have a health check, appears to be linked with people’s behavioral decisions and a subjective understanding of how well secondary prevention strategies work in mitigating disease.

People’s decisions about health check-ups can be observed from the viewpoint of the rational choice framework. Following Grossman’s health capital model [17–19], we see the health check-up as a derived demand for health investment, to potentially improve consumption (good health provides satisfaction) and production (individuals in good health earn more money) benefits. Financial literacy, being a rational decision-making tool, enables people to make informed economic, financial, and investment decisions [3, 20]. Therefore, endogenizing financial knowledge in the context of health check-ups can explain an individual’s rational behavioral decisions. Consequently, any uptake in health check-ups, motivated by financial literacy and education, can be regarded as a health investment that will boost health and decrease the rate of depreciation on the initial stock of health, caused by aging. Moreover, financially literate people are likely to enhance their consumption structure in relation to health insurance and health care, find better health services, and improve their health. Supporting this rational decision-making framework, Chan et al. [7] found that financial literacy influences preventive healthcare; James et al. [21] found that financial and health literacy influences health promoting behaviors and health status. Moreover, Meyer [22], Taylor et al. [23, 24], and Zheng et al. [25] found that financially literate people are more efficient at obtaining health services and maintaining better health status.

However, several complex and controversial issues surrounding health check-ups undermine the rationality explanation, which was found significant for other health-related behaviors in studies such as Chan et al. [7], Khan et al. [4] Watanapongvanich et al. [6], and Ono et al. [5]. Using 16 trials and 182,880 participants from various European countries, Krogsbøll et al. [26] concluded that general checks are unlikely to be effective in reducing morbidity or mortality, neither for overall health nor for cardiovascular disorders or cancer. A 2018 update to the review, led the authors to the same conclusion [11]. Other studies support this finding too [12, 27–29]. Many studies claim that preventive health screening harms patients in the form of overdiagnosis and unnecessary or risky treatments [30–32]. Dye [33] suggests that when decisions are made in pursuit of cure or prevention and do not coincide with expectations, they are categorized as “irrational.” Therefore, these studies hint that the concept of rationality cannot be applied to all medical circumstances.

As the role of financial literacy in health checkups is ambiguous, it would be noteworthy to observe how rational explanations of health check-ups operate, when healthcare efficiency is involved. This study aims to examine the association of financial literacy and financial education with the uptake of health check-ups among the Japanese population. The study contributes to the literature in two ways. First, examining the increasing disparity and slow uptake of preventive health check-ups from a rationality perspective, could provide new dimensions to better understand the prevailing health issues and the effectiveness of health programs in Japan. Second, to our knowledge, this is the first study that explicitly examines the association of financial literacy and financial education, with preventive health check-ups. Chan et al. [7] explored the role of financial literacy in preventive healthcare, which was an index of smoking, vaccination for flu, and health check-ups. However, this study was not solely dedicated to examining health check-up behavior from a rationality perspective with financial literacy. Moreover, Chan et al. [7] had a limited sample comprising 481 students from a regional university in Kentucky, US, and used different measures and methodologies for financial literacy. Despite these differences, Chan et al.’s findings are relevant to our study, and we have duly emphasized the connections when discussing our results.

## Methods

### Data

We used Osaka University’s Preference Parameters Study (PPS) for 2010 and 2011. The PPS was a panel survey conducted annually from 2003‒2013 that extracted information about Japanese people’s socioeconomic and demographic characteristics and preferences. We extracted data on financial literacy as the main independent variable and financial education as a second significant primary independent variable, from the 2010 dataset. From the 2011 onwards, we obtained data on health check-up behavior as a dependent variable, along with data on the control variables. Our sample focused on Japan’s middle-aged working population (40–64 years), for whom health check-ups were deemed more important. Furthermore, health check-ups and guidance, focusing on visceral fat obesity, target the insured and dependents aged 40 or over [34]. We combined the datasets using each respondent’s panel identification information; 3,420 observations remained after excluding samples with missing demographic and socioeconomic variables and unmatched data. Removing 1,212 individuals aged under 40 and over 64 yielded the final sample of 2,208 responses, which accounted for 44.75% of the total responses in the 2011 dataset (4,934 observations).

### Variables

A comprehensive overview of variable definitions is provided in this sub-section. To begin with, the dependent variable indicates the probability of getting a health check-up; that is, the chances that an individual had at least 1 type of health check-up in a year, excluding health check-ups organized by employers and schools. One of the questions in the 2011 PPS dataset enquires about the health check-up behavior of each respondent as such: “(Over the past 12 months) Have you had any health check-ups (excluding cancer examination, prenatal check, dental check-up, and medical treatment),” with six possible responses: “1 – Health check organized by local municipality,” “2 – Health check organized by your employee or labor union of your employee,” “3 – Health check organized by your school,” “4 – Medical check-up (other than or above 1–3),” “5 – Other,” “6 – I haven’t had any health checks in the last one year.” Through these responses, we created the dependent variable as a binary variable equal to 1, for any type of health check-up taken within a year besides those organized by the government, employers, and schools—this is because such check-ups are mandatory, so the idea of rationality does not apply.

We had two main variables of interest: financial literacy and financial education, which assess financial knowledge from the investment and savings behavior standpoints, respectively. The former is based on the three questions developed by Lusardi and Mitchell [35] provided in Appendix. These questions test an individual’s mathematical ability and the understanding of basic financial concepts such as interest rates, inflation, and risk diversification, which are critical in making sound investments. Owing to their simplicity and empirical support, several other studies have adopted these questions to measure financial literacy [3, 6, 36–39]. To quantify the financial literacy questions, we assigned a score of 1 for each correct answer and 0 for an incorrect answer. We took the equally weighted average scores of the three questions to obtain the financial literacy variable.

The other primary explanatory variable — financial education — was based on the following multiple-choice question in the PPS survey: “Did you receive any compulsory financial education when you were in elementary school?” We assigned a value of 1 if the respondent said “yes” and 0 if they said “no” or “do not know.” We included this option because Japan is the only country in Asia to offer financial education programs in its school curriculum [40]. These programs eventually influence financial stability risks and the corresponding preventive demand in the long term. Furthermore, financial education has no effect on financial literacy among Japanese people who previously received this type of teaching through the program [41]; hence, we evaluate both variables.

Other demographic and socioeconomic variables included gender, age, university degree, marital status, number of household members, children, employment status, household income, and household assets. We also controlled for risky health behaviors such as smoking, alcohol consumption, and gambling addiction. Finally, we included psychological variables such as myopic view of the future, current level of happiness, anxiety about health, and poor health status. A summarized version of the variables used, their types, definitions, and respective coding is provided in Table 1.

**Table 1:** Variable definitions

Variables	Definitions
Probability of getting a health check-up(overall)	Binary variable: 1 = any health check-ups taken within a year, 0 = otherwise (excluding health check-ups organized by employer and school)
Financial literacy	Continuous variable: average score for the number of current answers from three financial literacy questions
Financial education	Binary variable: 1 = received compulsory financial education at school, 0 = otherwise
Male	Binary variable: 1 = male, 0 = female
Age	Age of participants
University degree	Binary variable: 1 = obtained a university degree or higher, 0 = otherwise
Marriage	Binary variable: 1 = married, 0 = otherwise
Divorce	Binary variable: 1 = divorced, 0 = otherwise
Household size	The number of people living in the household
Children	Binary variable: 1 = have at least one child, 0 = otherwise
Unemployed	Binary variable: 1 = unemployed, 0 = otherwise
Household income	Annual earned income before taxes and with bonuses of the entire household in 2010 (JPY)
Log of household income	Log(household income)
Household assets	The balanced amount of financial assets (savings, stocks, insurance, etc.) of the entire household (JPY)
Log of household assets	Log(household assets)
Current smoker	Binary variable: 1 = smoke (occasionally–more than one pack a day), 0 = do not smoke (never smoke, hardly smoke, already quit smoking)
Current drinker	Binary variable: 1 = drink (sometimes–5 cans a day), 0 = do not drink (do not drink at all, hardly drink)
Frequent gambler	Binary variable: 1 = frequent gambler (once a week or more), 0 = otherwise
Myopic view of the future	Binary variable: 1 = agree/completely agree with “Since the future is uncertain, it is a waste to think about it,” 0 = otherwise
Current level of happiness	Continuous variable: percentage score from the question “Overall, how happy would you say you are currently?”
Anxiety about health	Binary variable: 1 = agree/completely agree with “I have anxiety about my health,” 0 = otherwise
Poor health status	Binary variable: 1 = describe current health status as poor, 0 = otherwise

### Methods

As our dependent variable was a binary variable, we performed a probit regression analysis. This was done to test our hypothesis that people with better financial literacy and financial education, are more likely to participate in non-mandatory preventive health checks. Equations 1 and 2 assess the impacts of financial literacy and financial education on health check-up behavior, individually. Model (3) incorporates both variables to assess their combined impact.

1 $${{\varvec{Y}}}_{{\varvec{i}}} = {\varvec{f}}({{\varvec{F}}{\varvec{L}}}_{{\varvec{i}}}, {{\varvec{X}}}_{{\varvec{i}}},{{\varvec{\varepsilon}}}_{{\varvec{i}}}),$$

2 $${{\varvec{Y}}}_{{\varvec{i}}}\boldsymbol{ }=\boldsymbol{ }{\varvec{f}}({{\varvec{F}}{\varvec{E}}}_{{\varvec{i}}},\boldsymbol{ }{{\varvec{X}}}_{{\varvec{i}}},{{\varvec{\varepsilon}}}_{{\varvec{i}}}),$$

3 $${{\varvec{Y}}}_{{\varvec{i}}}\boldsymbol{ }=\boldsymbol{ }{\varvec{f}}({{\varvec{F}}{\varvec{L}}}_{{\varvec{i}}},\boldsymbol{ }{{\varvec{F}}{\varvec{E}}}_{{\varvec{i}}},\boldsymbol{ }{{\varvec{X}}}_{{\varvec{i}}},{{\varvec{\varepsilon}}}_{{\varvec{i}}}),$$

where ${Y}_{i}$ indicates the preventive health check-up behavior of the $i$ th respondent, $FL$ represents the average financial literacy score, $FE$ is financial education status, $X$ is a vector of the respondents’ characteristics, and $\varepsilon$ is the error term. We utilized a probit model to predict health check-up behavior against financial literacy and financial education, after adjusting for socioeconomic and demographic characteristics. This model describes the probability of an event’s falling into one of the specified categories.

To identify and correct for high intercorrelations among two or more independent variables in all models, we conducted correlation and multicollinearity tests (available upon request). For example, individuals with a high level of education may have high financial knowledge, or those with a high net worth may have more financial knowledge because of their experience managing assets. The correlation matrix revealed a weak relationship between the relative movements of two variables across all models (substantially lower than 0.70). Furthermore, the variance inflation factor tests of the explanatory variables showed an insignificant presence of multicollinearity in all models.

Although potential endogeneity is a concern, we believe this is not going to affect our results, for several reasons. First, we included important factors that might influence individuals’ health checkup behavior. These comprise socio-economic, demographic, and health characteristics of individuals that are widely used in various health-economics studies to explain phenomena. Second, the theoretical background discussed in the introduction sections lays down the basis for how financial literacy could influence health check-up behavior and why reverse causality is less likely to occur. Finally, having a one-year lag in financial literacy scores compared with health check-up behavior and other data also minimizes the probability of reverse causality.

We created four models for Eqs. (1–3), each with a distinct control variable. We provided an example of our model requirements for Eq. (1) below. Equations (4), (5), (6), and (7), represent Models 1, 2, 3, and 4, respectively.

4 $$Probability of getting health {checkup}_{i} = \Phi \left( {\beta }_{0}+{\beta }_{1}{financial literacy}_{i}+{\beta }_{2}{male}_{i}+{\beta }_{3}{age}_{i}+ {\beta }_{4}university {degree}_{i}+{\beta }_{5}{marriage}_{i}+{\beta }_{6}{divorce}_{i}+{\beta }_{7}{household size}_{i}+{ \beta }_{8} {children}_{i}+{\beta }_{9}{unemployed}_{i}+{\beta }_{10}{logof household income}_{i}+{\beta }_{11}{logof household assets}_{i}\right),$$

5 $${Probability of getting health checkup}_{i}=\Phi ({ \beta }_{0}+{ \beta }_{1}{financial literacy}_{i}+{\beta }_{2}{male}_{i} +{\beta }_{3}{age}_{i}+{\beta }_{4}{university degree}_{i}+{\beta }_{5}{marriage}_{i}+{\beta }_{6} {divorce}_{i}+{\beta }_{7}{household size}_{i}+ {\beta }_{8}{children}_{i}+{\beta }_{9} {unemployed}_{i}+{ \beta }_{10}{logof household income}_{i}+{\beta }_{11}{logof household assets}_{i}+{\beta }_{12}{current smoker}_{i}+{\beta }_{13}{current drinker}_{i}+ {\beta }_{14}{frequent gamblers}_{i} ),$$

6 $${Probability of getting health checkup}_{i}= \Phi ({ \beta }_{0}+{\beta }_{1}{financial literacy}_{i}+{\beta }_{2}{male}_{i}+{\beta }_{3}{age}_{i} + {\beta }_{4}{university degree}_{i}+{\beta }_{5}{marriage}_{i}+{ \beta }_{6}{divorce}_{i}+{\beta }_{7}{household size}_{i}+ {\beta }_{8}{children}_{i} +{\beta }_{9}{unemployed}_{i}+{ \beta }_{10}{logof household income}_{i}+{\beta }_{11}{logof household assets}_{i} +{\beta }_{12}{current smoker}_{i}+{\beta }_{13}{current drinker}_{i} + {\beta }_{14}{frequent gamblers}_{i}+{ \beta }_{15}{myopic view of the future}_{i} ),$$

7 $${Probability of getting health checkup}_{i}= \Phi ( {\beta }_{0}+{ \beta }_{1}{financial literacy}_{i} +{\beta }_{2}{male}_{i} +{\beta }_{3}{age}_{i} +{\beta }_{4}{university degree}_{i} +{\beta }_{5}{marriage}_{i} + {\beta }_{6}{divorce}_{i} +{\beta }_{7}{household size}_{i}+{ \beta }_{8}{children}_{i} +{\beta }_{9}{unemployed}_{i} + {\beta }_{10}{logof household income}_{i} +{\beta }_{11}{logof household assets}_{i} +{\beta }_{12}{current smoker}_{i} +{\beta }_{13}{current drinker}_{i} + {\beta }_{14}{frequent gamblers}_{i} + {\beta }_{15}{myopic view of the future}_{i} +{\beta }_{16}{current level of the happiness}_{i} +{ \beta }_{17}{anxiety about health}_{i} +{\beta }_{18}{poor health status}_{i}).$$

## Results

### Descriptive statistics

In this study, statistics for some demographic variables, such as sex and mean age, correspond with the results of the 2021 population census. In the sample of 2208, about 48.8% were classified as men, and the average age of the Japanese population was 52.02 years. Statistics Bureau of Japan [42] in their census reported these levels at as 48.7% and 49 years, consecutively. This similarity, to some extent, signifies the national representativeness of the Japanese population in our study. The descriptive statistics of other demographic and socio-economic variables used in this study are also reported in Table 2. On average, only 31.75% (701) of the respondents had a health check-up in the prior year, indicating low participation in non-mandatory health checks and failure to meet targets set by MHLW. The average financial literacy score was 0.62, and approximately 17% of the respondents had received financial education in elementary school. Around 26.18% of the individuals held a university degree, 86.64% were married, and 4.53% were divorced. On average, 4 individuals lived in a household with a minimum and maximum household size, ranging from 1‒10. Approximately 87.41% of the respondents had at least one child, which is a typical characteristic of a developed and aging country, and only 2.36% were currently unemployed. In addition, the average annual household income and household asset net worth, was approximately JPY 6.89 million and JPY 13.50 million, respectively. Regarding risky behavior among the respondents, about 23.37% were current smokers, 48.64% were habituated to consuming alcohol, and 10.60% were frequent gamblers. Regarding the remaining psychological variables, only 15.81% of the respondents had a myopic view of the future, whereas 42.35% had anxiety about their health. Moreover, the respondent’s happiness level averaged at 63.51% and approximately 14.9% described their health status as being poor.

**Table 2:** Descriptive statistics

Variables	Mean	Std. Dev	Min	Max
**Dependent Variable**				
Probability of getting health check-up(overall)	0.3175	0.4656	0	1
**Main Independent Variables**				
Financial literacy	0.6185	0.3314	0	1
Financial education	0.1694	0.3752	0	1
**Other Independent Variables**				
Male	0.4882	0.5000	0	1
Age	52.0263	7.1732	40	64
University degree	0.2618	0.4397	0	1
Marriage	0.8664	0.3403	0	1
Divorce	0.0453	0.2080	0	1
Household size	3.6141	1.4012	1	10
Children	0.8741	0.3318	0	1
Unemployment	0.0236	0.1517	0	1
Household income	6,888,813	3,896,541	1,000,000	20,000,000
Log of household income	15.5784	0.6082	13.8155	16.8112
Household asset	13,500,000	17,200,000	2,500,000	100,000,000
Log of household asset	15.8645	1.0026	14.7318	18.4207
Current smoker	0.2337	0.4233	0	1
Current drinker	0.4864	0.4999	0	1
Frequent gambler	0.1060	0.3079	0	1
Myopic view of the future	0.1581	0.3649	0	1
Current level of happiness	0.6351	0.1817	0	1
Anxiety about health	0.4235	0.4942	0	1
Health status	0.0149	0.1214	0	1
Observations	2208			

To compare the average values of the two data sets and determine if they came from the same population, a t-test was conducted for the probability of having a health check-up, distributed by age group, demographic characteristics, and risky health behavior. The results of the above test are provided in Tables 3, 4, and 5 respectively, along with the test statistics and significance levels.

**Table 3:** Distribution of probability of getting health check-up by age group

Probability of getting health check-up	Age		Total
	40–50	51–64	
0	752	755	1507
	76.58%	61.58%	68.25%
1	230	471	701
	23.42%	38.42%	31.75%
Total	982	1226	2208
	100%	100%	100%
Mean Difference	t = -7.6173***		

Note: ***p < 0.01

**Table 4:** Distribution of the probability of having a health check-up by demographic characteristic

Probability of getting health check-up	Gender		Education		Unemployed		Total
	Female	Male	Below University degree	Above university degree	No	Yes	
0	712	795	1096	411	1476	31	1507
	63.01%	73.75%	67.24%	71.11%	68.46%	59.62%	68.25%
1	418	283	534	167	680	21	701
	36.99%	26.25%	32.76%	28.89%	31.54%	40.38%	31.75%
Total	1130	1078	1630	578	2156	52	2208
	100%	100%	100%	100%	100%	100%	100%
Mean Difference	t = 5.4526***		t = 1.7168*		t = -1.3539		

Note: ***p < 0.01

^*^p < 0.10

**Table 5:** Distribution of the probability of having a health check-up by risky health behavior

Probability of getting health check-up	Current smoker		Current drinker		Frequent gambler		Total
	No	Yes	No	Yes	No	Yes	
0	1098	409	747	760	1331	176	1507
	64.89%	79.26%	65.87%	70.76%	67.43%	75.21%	68.25%
1	594	107	387	314	643	58	701
	35.11%	20.74%	34.13%	29.24%	32.57%	24.79%	31.75%
Total	1692	516	1134	1074	1974	234	2208
	100%	100%	100%	100%	100%	100%	100%
Mean Difference	t = 6.1888***		t = 2.4697**		t = 2.4217**		

Note: ***p < 0.01

^**^p < 0.05

In the distribution for probability of getting health check-up by age group, we note that Table 3 shows a considerable difference in health check-up behavior of age groups 40–50 and 51–64, with the former showing a lower tendency to have check-ups (23% vs. 38%). All other things being equal, this vast difference implies that age could have a sizable effect on health check-up behavior.

Similarly, in the distribution for the probability of obtaining a health check-up by demographic characteristics, Table 4 establishes a notable difference in health check-up behavior by gender and educational variables. However, it posits insignificant disparities by employment status. Women (36.99%) had more check-ups than men (26.25%) at a 1% significance level. Notably, the proportion of people with higher qualifications (above university degree) who had had a health check-up was almost 4% lower than that of people with lower qualifications at the 10% level of significance. Similarly, we found an insignificant health check-up gap between the unemployed (40.38%) and employed (31.54%). All other things being equal, the gaps resulting from gender and education, imply that these variables could have a substantial effect on health check-up behavior.

Finally, in the distribution for the probability of getting a health check-up according to risky health behaviors, Table 5 establishes a dramatic heterogeneity in health check-up behavior as categorised by smoking, alcohol consumption, and frequent gambling. More specifically, the proportion of respondents who had a health check-up and smoked (20.74%) was substantially lower than the proportion who were non-smokers (35.11%) at a 1% significance level. Similarly, alcohol drinkers had fewer check-ups (29.24%) than non-drinkers (34.13%), at a 5% significance level. Our results for gambling behavior were similar. All other things being equal, this vast difference implies that smoking, alcohol consumption, and gambling could have a sizable effect on health check-up behavior.

### Regression analysis

The regression results in Tables 6 and 7 show the relationship of the probability of having a health check-up with financial literacy, financial education, and both together. Model 1.1 contains the main explanatory variable and the demographic characteristics. Model 1.2 incorporates the other variables related to risky health behavior into Model 1.1. Model 1.3 combines Models 1.2 and 1.3, with the addition of myopic view of the future. The final model, Model 1.4, adds current level of happiness, anxiety about health, and poor health status as psychological variables. Models 2.1–2.4 and 3.1–3.4 use the same control variables.

**Table 6:** Probit model regression results with financial literacy or financial education as the main explanatory variable

**Variables**	**Dependent variable: Probability of getting health-check-up**							
	**Financial literacy as main explanatory variable**				**Financial education as main explanatory variable**			
	**Model 1.1**	**Model 1.2**	**Model 1.3**	**Model 1.4**	**Model 2.1**	**Model 2.2**	**Model 2.3**	**Model 2.4**
Financial literacy	0.0040	-0.0103	-0.0113	-0.0225				
	(0.0902)	(0.0905)	(0.0905)	(0.0908)				
Financial education					0.0239	0.0203	0.0195	0.0231
					(0.0756)	(0.0756)	(0.0756)	(0.0759)
Male	-0.3273***	-0.2354***	-0.2361***	-0.2305***	-0.3263***	-0.2360***	-0.2368***	-0.2322***
	(0.0614)	(0.0687)	(0.0687)	(0.0689)	(0.0608)	(0.0681)	(0.0681)	(0.0683)
Age	0.0274***	0.0273***	0.0274***	0.0281***	0.0273***	0.0271***	0.0273***	0.0279***
	(0.0046)	(0.0047)	(0.0047)	(0.0047)	(0.0047)	(0.0047)	(0.0047)	(0.0047)
University degree	-0.0063	-0.0294	-0.0306	-0.0378	-0.0067	-0.0308	-0.0321	-0.0403
	(0.0716)	(0.0724)	(0.0724)	(0.0725)	(0.0709)	(0.0717)	(0.0717)	(0.0719)
Marriage	0.3622***	0.3521***	0.3528***	0.3234**	0.3606***	0.3505***	0.3513***	0.3215**
	(0.1248)	(0.1260)	(0.1259)	(0.1268)	(0.1249)	(0.1261)	(0.1260)	(0.1270)
Divorce	0.1845	0.2287	0.2312	0.2020	0.1848	0.2285	0.2309	0.2013
	(0.1775)	(0.1795)	(0.1796)	(0.1801)	(0.1773)	(0.1793)	(0.1794)	(0.1799)
Household size	-0.0099	-0.0104	-0.0105	-0.0080	-0.0101	-0.0104	-0.0105	-0.0079
	(0.0247)	(0.0249)	(0.0249)	(0.0249)	(0.0246)	(0.0248)	(0.0248)	(0.0249)
Children	-0.1424	-0.1570	-0.1571	-0.1586	-0.1429	-0.1570	-0.1570	-0.1582
	(0.1058)	(0.1070)	(0.1070)	(0.1074)	(0.1058)	(0.1069)	(0.1070)	(0.1073)
Unemployed	0.3366*	0.3243*	0.3286*	0.3579*	0.3368*	0.3247*	0.3290*	0.3585**
	(0.1815)	(0.1806)	(0.1809)	(0.1827)	(0.1814)	(0.1805)	(0.1808)	(0.1826)
Log of household income	-0.0366	-0.0341	-0.0349	-0.0576	-0.0364	-0.0347	-0.0355	-0.0588
	(0.0545)	(0.0549)	(0.0549)	(0.0562)	(0.0543)	(0.0548)	(0.0548)	(0.0561)
Log of household assets	0.1229***	0.1106***	0.1099***	0.1025***	0.1228***	0.1099***	0.1091***	0.1011***
	(0.0315)	(0.0318)	(0.0319)	(0.0321)	(0.0312)	(0.0315)	(0.0316)	(0.0319)
Current smoker		-0.2946***	-0.2937***	-0.2782***		-0.2944***	-0.2934***	-0.2777***
		(0.0752)	(0.0752)	(0.0754)		(0.0751)	(0.0751)	(0.0754)
Current drinker		-0.0101	-0.0111	-0.0130		-0.0099	-0.0109	-0.0129
		(0.0614)	(0.0614)	(0.0616)		(0.0614)	(0.0614)	(0.0616)
Frequent gambler		-0.0876	-0.0867	-0.0865		-0.0860	-0.0851	-0.0845
		(0.0983)	(0.0983)	(0.0982)		(0.0984)	(0.0983)	(0.0983)
Myopic view of the future			-0.0409	-0.0380			-0.0403	-0.0371
			(0.0780)	(0.0780)			(0.0780)	(0.0779)
Current level of happiness				0.3324*				0.3324*
				(0.1742)				(0.1741)
Anxiety about health				-0.0520				-0.0514
				(0.0597)				(0.0597)
Poor health status				0.1653				0.1630
				(0.2220)				(0.2217)
Constant	-3.3155***	-3.0804***	-3.0552***	-2.7926***	-3.3110***	-3.0612***	-3.0355***	-2.7597***
	(0.8557)	(0.8619)	(0.8636)	(0.8745)	(0.8521)	(0.8593)	(0.8612)	(0.8726)
Observations	2,208	2,208	2,208	2,208	2,208	2,208	2,208	2,208
Log likelihood	-1316	-1308	-1308	-1305	-1316	-1308	-1308	-1305
Chi2 statistics	122.4	135.8	136.5	144.8	122.3	135.6	136.3	144.4
p-value	0	0	0	0	0	0	0	0

Robust standard errors in parentheses *** *p* < 0.01, ** *p* < 0.05, * *p* < 0.1

**Table 7:** Probit model regression results with financial literacy and financial education as the main explanatory variables

Variables	Dependent variable: Probability of getting health-check-up			
	**Model 3.1**	**Model 3.2**	**Model 3.3**	**Model 3.4**
Financial literacy	0.0036	-0.0106	-0.0116	-0.0229
	(0.0902)	(0.0905)	(0.0905)	(0.0909)
Financial education	0.0238	0.0204	0.0197	0.0234
	(0.0756)	(0.0756)	(0.0757)	(0.0759)
Male	-0.3266***	-0.2350***	-0.2357***	-0.2299***
	(0.0614)	(0.0687)	(0.0687)	(0.0688)
Age	0.0273***	0.0271***	0.0273***	0.0280***
	(0.0047)	(0.0047)	(0.0047)	(0.0047)
University degree	-0.0070	-0.0299	-0.0311	-0.0384
	(0.0715)	(0.0723)	(0.0723)	(0.0725)
Marriage	0.3605***	0.3507***	0.3515***	0.3217**
	(0.1249)	(0.1261)	(0.1261)	(0.1270)
Divorce	0.1847	0.2289	0.2313	0.2020
	(0.1773)	(0.1794)	(0.1795)	(0.1800)
Household size	-0.0101	-0.0106	-0.0107	-0.0082
	(0.0247)	(0.0249)	(0.0249)	(0.0249)
Children	-0.1428	-0.1574	-0.1574	-0.1591
	(0.1058)	(0.1069)	(0.1070)	(0.1074)
Unemployed	0.3369*	0.3246*	0.3289*	0.3585**
	(0.1814)	(0.1805)	(0.1808)	(0.1826)
Log of household income	-0.0366	-0.0342	-0.0349	-0.0578
	(0.0545)	(0.0549)	(0.0549)	(0.0562)
Log of household assets	0.1226***	0.1105***	0.1097***	0.1024***
	(0.0315)	(0.0318)	(0.0319)	(0.0321)
Current smoker		-0.2947***	-0.2938***	-0.2784***
		(0.0752)	(0.0752)	(0.0755)
Current drinker		-0.0098	-0.0109	-0.0128
		(0.0614)	(0.0614)	(0.0616)
Frequent gambler		-0.0860	-0.0852	-0.0846
		(0.0984)	(0.0983)	(0.0983)
Myopic view of the future			-0.0405	-0.0375
			(0.0780)	(0.0780)
Current level of happiness				0.3336*
				(0.1742)
Anxiety about health				-0.0522
				(0.0597)
Poor health status				0.1627
				(0.2220)
Constant	-3.3068***	-3.0732***	-3.0486***	-2.7837***
	(0.8561)	(0.8623)	(0.8640)	(0.8750)
Observations	2,208	2,208	2,208	2,208
Log likelihood	-1316	-1308	-1308	-1305
Chi2 statistics	122.4	135.7	136.4	144.7
p-value	0	0	0	0

Robust standard errors in parentheses

^***^ *p* < 0.01

^**^ *p *< 0.05

^*^ *p* < 0.1

The results in Table 6 reveal the insignificant role of financial literacy in health check-up behavior in Japan. Similarly, financial education is also insignificant across all models. The results in Table 7, which contain both the explanatory variables, indicate no significant variations in the significance of the estimated parameters, compared to those in Table 6. This finding suggests that financial literacy and financial education have statistically insignificant effects on preventive health check-up behavior in Japan. Therefore, owing to the robustness and consistency in the correlation of the main explanatory variables in Tables 6 and 7, we focused on the results in Table 7, where all the control variables have similar signs and significance levels, across all models. We found that being older, married, or unemployed, and having higher levels of household assets and higher subjective feelings of happiness had positive relationships of varying significance with the probability of having a health check-up, at the 1% significance level. Being male and smoking were negatively related to having a health check-up at the 10% significance level. Moreover, having a university degree, being divorced, having children, larger/smaller households, household income, drinking and gambling behavior, myopic view of the future, anxiety about health, and poor health status were not significantly associated with health check-up behavior.

## Discussion

Unlike the study by Chan et al. [7] which reported a positive association between financial literacy and the use of preventive healthcare in the US, our study found that financial literacy and financial education had no association with health check-up behavior in Japan. Since our study has significant differences from that of Chan et al., [7] direct comparisons of results are difficult. We explain the insignificant association of financial literacy and financial education with health check-up behavior, from the viewpoint of the rationality explanation and some socio-medical factors that reveal the level of healthcare efficiency. The insignificant association is supported by the fact that the rationality of medical check-ups is complex, and requires comprehensive and multifaceted assessment criteria [43]. First, how financially literate people perceive health check-ups in terms of their overall benefits in detecting and solving risk factors for various diseases could mitigate the causal relationship between financial literacy and the rational choice to have a health check-up [44]. In Japan, various secondary prevention strategies are offered through routine health check-ups; however, it is unclear if additional health checkups are valuable to the system in terms of population health and cost-saving [13]. Dye [33] argues that routine health checks are typically used or offered to those who need them the least, as they are mostly at a lower risk of illness, thus giving a poor return on investment. Moreover, a good health check-up is characterized by high positive and negative predictive values, and are of “no use” if they do not provide this certainty [45]. Studies [11, 12, 26–29] have concluded that health checks are ineffective in reducing morbidity or mortality. In some instances, they have resulted in overdiagnosis and unnecessary or risky treatments [30–32]. This causes distrust in healthcare quality and unfavorable perceptions of public health status [46], weakening the rationality aspect of health check-ups [47]. Generally, when preventive or treatment options do not meet expectations, they are labeled as irrational [33]. Yamada and Yamada [48] argued that a health check-up is a time-consuming health input, as people sacrifice valuable work hours or days to get their health checked. Therefore, it is safe to say that respondents with higher levels of financial literacy or financial education are rational in their perception about the limitations of screening. Their decision of non-participation is subjectively rational and can be explained through their practice of “trading-off” positive and negative aspects of health-promoting behavior, such as health check-ups [49]. Based on this evidence, the financial-literacy-induced rationality theory of health check-ups is challenged by this study.

In addition, we found that demographic factors have considerable influences on health check-up decisions. Specifically, being male, older, and married were significantly related to health check-up behavior, whereas household size, being divorced, and having children, were not. Moreover, men showed a lower tendency to have preventive health check-ups than women. Our results strongly agree with those of a prior study [50], suggesting that this could possibly explain why men, in general, have poorer health [51]. Several other studies also indicate that women are more health-conscious than men and are more likely to have health check-ups [52, 53]. Moreover, as people get older, they are more likely to have health check-ups. Given that Japan has an aging population with 28.7% of the population being 65 or older, health check-ups would be more common among the older population, as they are at a higher risk of developing non-communicable diseases [54]. This result is consistent with the finding that the older population requires more healthcare services, as comorbidities increase with age [55, 56]. Regarding marital status variables, although divorce has an insignificant impact on health check-up behavior, marriage showed a considerably positive association. This finding was consistent with Korkeila et al. [57], who showed that divorced and widowed persons were more hesitant toward positive health behavior. Hoebel et al. [58] and Wilson et al. [59] found that married individuals were more willing to participate in regular health checks. This can be attributed to greater attention to health symptoms and higher awareness of preventive measures among those living with a partner [60]. Moreover, we found that socioeconomic variables such as unemployment and household assets were significantly related to health check-up behavior, whereas university degree and household income were not. For the unemployment variable, a significant and positive link with health check-up behavior was an unexpected finding, as it did not align with many other studies [8, 61, 62]. One justification for this may be that as our study excludes those participating in mandatory health check programs organized at school or work, all other non-mandatory forms of health check-ups would be greatly accessible to unemployed people, because they would have more time and energy. However, we found a significant and positive association for household assets, but not household income. One major cause for this is that wealth is more closely related to health status than income [63], with more consistent effects [64].

Of all risky health behavior variables such as smoking, alcohol consumption. and gambling addiction, only smoking had a significant impact on health checkup behavior in Japan. Smoking reduces the chance of accessing health checks, which corresponds with the findings of previous studies [8, 58, 59]. This is because participating in risky behaviors that individuals know are harmful, makes them reluctant to have check-ups. Meanwhile, the insignificant and negative association of alcohol consumption could arise from the unwillingness of excessive alcohol drinkers to have their possible risk factors revealed or their feeling of good health disturbed [49].

For the remaining psychological variables, apart from current level of happiness, variables such as myopic view of the future, anxiety about health, and poor health status had an insignificant effect on health check-up behavior. The positive and significant association of the current level of happiness with health check-up behavior is consistent with Goel et al. [65]. They suggest that people who are less satisfied with life, are more likely to come from poorer socioeconomic backgrounds. Hence, they are more likely to refuse health screenings because of the high expense of healthcare consumption, at a later stage.

### Limitations

This study has several limitations. First, we evaluated only the 2011 PPS because it offered information on the likelihood of obtaining a health check-up in the previous year. As our study does not provide longitudinal data on the relationship between financial literacy, financial education, and preventive health checks, the causality of the insignificant relationship could possibly be strengthened in favor of our initial hypothesis. Thus, future studies should examine this association more in-depth. Second, we excluded health check-ups organized by the government, employers or schools, to measure rational behavior toward health check-ups effectively, by avoiding the bias of mandatory health check-ups. However, excluding such health check-ups may undermine the overall significance of the model. Hence, future studies should evaluate mandatory health check-ups against other control variables. Finally, we used only three questions to assess financial literacy and one question to assess financial education, even though it is a common method in similar research.

## Conclusion

Previous studies provide evidence on the role of financial literacy as a rational decision-making tool to reduce the chance of smoking [4] and gambling [6] and to increase the probability of frequent exercise [6] among the Japanese population. Hence, from the viewpoint of rational choice, we hypothesized a positive association between financial literacy and health check-up behavior among the Japanese population. However, our findings reveal that financial literacy and financial education have only statistically insignificant associations with people’s decision to participate in health checks. Our study suggests that some socio-medical factors, such as ineffectiveness of health check-ups in reducing morbidity or mortality and instances of over diagnosis and risky treatments, result in inefficiency in healthcare. This result may lessen the impact of financial literacy and financial education on health check-ups. Healthcare authorities may need to address any related socio-medical issues that exist in Japan’s health check-up program in order to influence people’s objective rationality and motivate them to engage in health check-up. This may be achieved by developing comprehensive and multifaceted assessment criteria for health check-ups and possibly testing some specific types of secondary prevention strategies to increase their effectiveness and efficiency.

## Data Availability

Data is available and can be accessed from https://www.iser.osaka-u.ac.jp/survey_data/top_eng.html

## Appendix

- Suppose you had 10,000 JPY in a savings account and the interest rate is 2% per year, and you never withdraw money or interest. After 5 years, how much would you have in this account in total?
  - More than ¥10,200
  - Exactly ¥10,200
  - Do not know
  - Refuse to answer
- Imagine that the interest rate on your savings account is 1% per year and inflation is 2% per year. After 1 year, how much would you be able to buy with the money in this account?
  - More than today
  - Exactly the same
  - Less than today
  - Do not know
  - Refuse to answer
- Please indicate whether the following statement is true or false. “Buying a company stock usually provides a safer return than a stock mutual fund”
  - True
  - False
  - Do not know

Refuse to answer.
